# Intelligent classification of computer vulnerabilities and network security management system: Combining memristor neural network and improved TCNN model

**DOI:** 10.1371/journal.pone.0318075

**Published:** 2025-01-27

**Authors:** Zhenhui Liu

**Affiliations:** 1 School of Information and Communication Engineering, Beijing University of Information Science and Technology, Bei Jing City, China; 2 Aviation Industry Information Center, Bei Jing City, China; Guangdong University of Petrochemical Technology, CHINA

## Abstract

To enhance the intelligent classification of computer vulnerabilities and improve the efficiency and accuracy of network security management, this study delves into the application of a comprehensive classification system that integrates the Memristor Neural Network (MNN) and an improved Temporal Convolutional Neural Network (TCNN) in network security management. This system not only focuses on the precise classification of vulnerability data but also emphasizes its core role in strengthening the network security management framework. Firstly, the study designs and implements a neural network model based on memristors. The MNN, by simulating the memory effect of biological neurons, effectively captures the complex nonlinear relationships within vulnerability data, thereby enhancing the data insight capabilities of the network security management system. Subsequently, structural optimization and parameter adjustments are made to the TCNN model, incorporating residual connections and attention mechanisms to improve its classification performance, making it more adaptable to the dynamically changing network security environment. Through data preprocessing, feature extraction, and model training, this study conducts experimental validation on a public vulnerability dataset. The experimental results indicate that: The MNN model demonstrates excellent performance across evaluation metrics such as Accuracy (ACC), Precision (P), Recall (R), and F1 Score, achieving an ACC of 89.5%, P of 90.2%, R of 88.7%, and F1 of 89.4%. The improved TCNN model shows even more outstanding performance on the aforementioned evaluation metrics. After structural optimization and parameter adjustments, the TCNN model’s ACC increases to 93.8%, significantly higher than the MNN model. The P value also improves, reaching 91.5%, indicating enhanced capability in reducing false positives and improving vulnerability identification accuracy. The integrated classification system, leveraging the strengths of both the MNN and improved TCNN models, achieves an ACC of 95.2%. This improvement not only demonstrates the system’s superior capability in accurately classifying vulnerability data but also proves the synergistic effect of MNN and TCNN models in addressing complex network security environments. The comprehensive classification system proposed in this study significantly enhances the classification performance of computer vulnerabilities, providing robust technical support for network security management. The system exhibits higher accuracy and stability in handling complex vulnerability datasets, making it highly valuable for practical applications and research.

## Introduction

In the context of the rapid development of information technology and the widespread adoption of the internet, computer systems and network security become central concerns. Frequent network attacks and data breaches cause significant harm to both the economy and personal privacy, highlighting the increasing importance of cybersecurity measures. Currently, the number and complexity of computer vulnerabilities rise continuously [1]. Traditional vulnerability detection and classification methods, which rely on manual efforts and predefined rule matching, are increasingly inadequate for handling large-scale and dynamically changing vulnerability data. The causes of vulnerabilities mainly involve three key areas: network protocols, application software systems, and misconfigurations. At the network protocol level, while the TCP/IP protocol provides the fundamental framework for network interconnection, it was developed in the relatively simple early days of networking and has inherent security flaws [2]. For example, the "Heartbleed" vulnerability, which exists in the OpenSSL heartbeat extension, allows attackers to craft malicious heartbeat packets to illegally read server memory data, stealing sensitive information such as private keys and user credentials. This severely threatens the security of network services relying on SSL/TLS encryption, affecting various fields, including financial transactions and e-commerce [3]. It undermines data confidentiality and integrity, emphasizing the serious erosion of network security foundations due to protocol vulnerabilities and the urgent need for building more reliable encryption communication mechanisms and comprehensive vulnerability monitoring systems. Regarding application software system vulnerabilities, risks can exist from the operating system’s underlying layers to upper-layer applications. Take the "WannaCry" ransomware as an example, which exploits a vulnerability in the SMB protocol of Windows systems to spread rapidly [4]. A large number of computers, which have not updated their patches in time, get infected, and critical business files of enterprises get encrypted for ransom. This results in business interruptions and severe economic losses, indicating that application software system vulnerabilities pose a fatal threat to business continuity and data availability. This prompts companies to enhance software update management, optimize vulnerability patching processes, and establish security development standards. In the area of misconfiguration vulnerabilities, when security policies are incorrectly configured, administrators struggle to effectively identify and resolve vulnerability issues. For example, excessive server permissions or improper access control list settings can provide malicious attackers with opportunities to easily access sensitive resources or perform malicious operations, severely weakening system security and potentially leading to system crashes or data breaches. This highlights the necessity of accurately configuring security policies, strengthening configuration audit mechanisms, and improving administrators’ security awareness to establish a solid network security defense against the complex and ever-changing risk of vulnerabilities [5].

In the field of automatic classification of computer vulnerabilities, Liu et al. (2022) proposed a novel sentiment classification model called Dependency Tree Graph Convolutional Network (DTGCN). This model combined Chinese grammatical dependency trees with Graph Convolutional Network (GCN), effectively utilizing grammatical information to improve the accuracy of Chinese text sentiment classification. Experimental results showed that the DTGCN model achieved an accuracy of 90.51% and a Recall (R) of 90.34% on the dataset, significantly improving over traditional models like Long Short-Term Memory (LSTM), demonstrating its advantage in uncovering complex internal relationships within texts, and highlighting its important application value in social network sentiment analysis [6]. Wang et al. (2023) proposed an automatic software vulnerability classification algorithm based on weighted word vectors and integrated neural networks. This algorithm generated low-dimensional weighted word vectors using the Term Frequency-Inverse Document Frequency (TF-IDF) algorithm and combined the Text Convolutional Neural Network-Bidirectional Gated Recurrent Unit (TextCNN-BiGRU) model. The Convolutional Neural Networks (CNNs) and BiGRU network respectively extracted local and global features, which were then fused and classified using a Softmax classifier. Experimental results validated the algorithm’s superiority in multiple metrics, including Accuracy (ACC) and F1 score, demonstrating its effectiveness and stability in software vulnerability data classification [7]. Wan et al. (2023) explored the application of web crawlers in detecting computer software security vulnerabilities. They analyzed the key role of web crawlers in data collection and information analysis, particularly the security risks and challenges encountered when processing large amounts of open information on the internet. They emphasized the effective detection of software security vulnerabilities through web crawler technology, providing new research perspectives and technical support for enhancing network security management systems [8]. Do Xuan et al. (2023) proposed a new source code vulnerability detection method based on intelligent cognitive computing. This method combined source code embedding, feature learning, data resampling, and classification models, utilizing various data extraction techniques to achieve the detection and classification of source code vulnerabilities [9].

The Memristor Neural Network (MNN), as an emerging neural network model, effectively captures complex nonlinear relationships in data by simulating the memory effect of biological neurons. The memristor, serving as the core component of MNN, has a memory function that can record historical input signals. This enables MNN to show significant potential in handling time-series data and large-scale complex pattern recognition tasks. In the field of network security, the introduction of MNN provides a new approach and methodology for addressing complex vulnerability classification problems. Additionally, CNNs have achieved remarkable results in image processing and text classification. Particularly, the Text Convolutional Neural Network (TextCNN) excels in feature extraction and classification of text data. TextCNN can automatically extract meaningful features from text through convolution operations and capture n-gram features of different lengths using convolutional kernels, demonstrating excellent performance in text classification tasks. This capability allows TextCNN to play a crucial role in processing descriptions of computer vulnerabilities and related textual information.

This study proposes integrating the MNN with an improved TextCNN model to construct an efficient comprehensive classification system. This system is expected to make groundbreaking advancements in the intelligent classification of computer vulnerabilities and network security management. By combining the strengths of both models, the new classification system can achieve higher accuracy and stability in handling complex vulnerability data, thereby enhancing the overall efficiency and effectiveness of network security management.

## Intelligent classification methods for computer vulnerabilities

### Computer vulnerabilities

A vulnerability refers to inherent defects in software and hardware, also known as weaknesses or flaws [10]. The causes of vulnerabilities are diverse and mainly include the following categories: (1) Network protocol vulnerabilities: Transmission Control Protocol/Internet Protocol (TCP/IP) protocols, developed in a trusted environment, focus on connectivity and openness but lack in security, resulting in numerous issues for services based on these protocols. (2) Application software system vulnerabilities: Vulnerabilities can occur at various levels of a system. Flaws in the design of either the operating system or installed programs can lead to system weaknesses [11]. (3) Misconfiguration vulnerabilities: When security policy configurations are flawed, administrators may struggle to quickly identify and resolve vulnerability issues, rendering security policies ineffective during system operation.

To effectively analyze, assess, and manage vulnerabilities, researchers have created various vulnerability databases to record all known vulnerabilities for study. A vulnerability database is a platform for storing, maintaining, and disseminating information about vulnerabilities found in actual computer systems, enabling security assessments and vulnerability management [12]. Additionally, each vulnerability is stored with a unique identifier, facilitating information sharing. The National Vulnerability Database (NVD) is one of the most widely used and well-known vulnerability databases, managed by the US National Institute of Standards and Technology (NIST). NVD provides data according to the Security Content Automation Protocol (SCAP) standards, which include the Common Vulnerabilities and Exposures (CVE) dictionary, listing all uniquely identified vulnerabilities and categorized by Common Weakness Enumeration (CWE). CVE also includes the Common Vulnerability Scoring System (CVSS) to assess the impact and severity of vulnerabilities [13]. The NVD website offers vulnerability data downloads in Extensible Markup Language (XML) format, covering data from both before 2002 and annually since 2003. The data collected by NVD are unique and highly compatible, allowing researchers to use them for experimental studies.

The China NVD of Information Security (CNNVD) is established in October 2009 by the China Information Technology Security Evaluation Center with support from national special funds, aiming to maintain national information security and manage data. CNNVD collects security vulnerabilities in operating systems and potential issues in network equipment through independent discovery, social submissions, collaborative sharing, web collection, and technical detection. It analyzes, verifies, warns, reports, and remedies these vulnerabilities [14]. CNNVD collaborates with government departments, industry users, security vendors, universities, and research institutions to standardize vulnerability assessment methods, eliminate information barriers, and provide assurance and foundational services for China’s information security [15]. The vulnerabilities addressed span various sectors including government, finance, transportation, industrial control, and healthcare. They safeguard infrastructure and provide data and theoretical support for industry development, playing a significant role in information security analysis.

### TCNN model

The TextCNN is a text classification method based on CNN, widely used in the field of Natural Language Processing (NLP). Also known as TextCNN, it enhances text classification performance by automatically extracting local features from text data through convolution operations [16].

**Fig 1:**
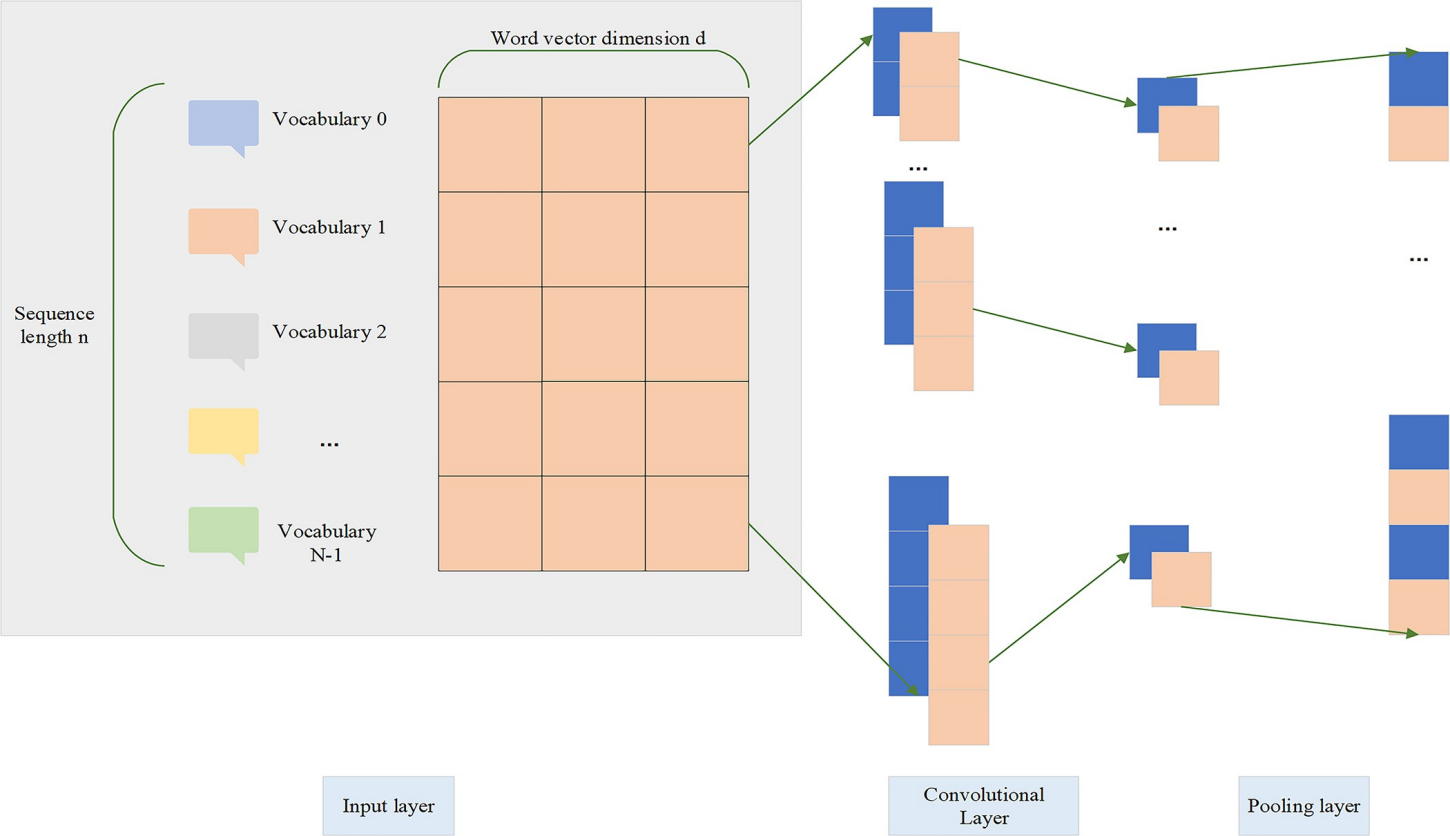
TextCNN structure.

The core idea of TextCNN is to use convolutional layers to apply sliding windows over text data to capture local contextual information [17]. Specifically, TextCNN achieves text classification through the following steps: (1) Input Layer: The input text is converted into word embeddings. These word embeddings are usually pre-trained, such as Word2Vec or GloVe, but can also be randomly initialized and updated during training. The length of the input text is typically fixed, with excess parts truncated and insufficient parts padded [18]. (2) Convolutional Layer: Multiple filters with different window sizes (e.g., 2-gram, 3-gram, 4-gram, etc.) are applied to the word embeddings representation of the text to extract local features of different scales. The convolution operation acts as a sliding window extracting local n-gram features. (3) Activation Function: The feature maps generated by the convolution are processed by a nonlinear activation function (e.g., ReLU, Rectified Linear Unit) to introduce nonlinearity and enhance the model’s expressiveness [19]. (4) Pooling Layer: A pooling operation (e.g., max pooling) is applied to the feature maps output by the convolutional layer to reduce their dimensionality while retaining the most important features. Pooling helps extract global features of the text and reduces computational complexity and overfitting risk [20–22]. (5) Fully Connected Layer: The pooled feature vectors are fed into a fully connected layer for further combination and abstraction, ultimately generating a fixed-length feature representation. (6) Output Layer: The output of the fully connected layer is mapped to a probability distribution over classes using the softmax function, completing the text classification task. The structure of TextCNN is shown in Fig 1.

In the convolutional layer, filters of various sizes extract features from the input matrix. A filter is typically defined as ***ω***∈***R***^***h×d***^, where ***h*** is the height of the filter, a hyperparameter usually set between 2 and 5, and ***d*** is the width of the filter, which equals the length of the word embeddings. By performing convolution operations with the filter at different window positions of the vulnerability text matrix, a feature map ***S***_***j***_***ϵR***^***n*−*h*+1**^ is obtained, where ***n*** represents the total number of words in the text. Convolution operations with different filter sizes produce feature maps of various dimensions. In CNNs architectures, due to the varying sizes of feature maps generated by filters of different dimensions, pooling operations are essential to achieve uniform feature dimensions. Common pooling functions include max pooling and average pooling. The reason for selecting max pooling in this study is based on a deep analysis of the characteristics of the vulnerability classification task and a precise consideration of the feature extraction effects [23]. During the feature extraction process, the differences in filter sizes have a significant impact on the extraction results. Smaller-sized filters can sensitively capture subtle local features in textual data, such as specific keyword combinations or syntactic structures, which help accurately identify vulnerability types with significant local features. Larger-sized filters are better at capturing semantic features in broader contexts, which facilitates understanding the overall meaning and potential correlations of vulnerability description texts from a macro perspective, catering to the needs of vulnerability classification in complex semantic situations. The comprehensive use of filters of different sizes can fully mine the rich features of vulnerability texts, but this also leads to inconsistent feature map dimensions. In this context, max pooling demonstrates its advantages. The core principle of max pooling is to precisely select the maximum value from the feature map output by the filter to capture the most critical and significant features. Compared to average pooling, max pooling focuses on the most discriminative feature elements in the text, efficiently retaining and enhancing the local extreme features that play a key role in vulnerability classification. This avoids the dilution of important information due to averaging, aligning with the requirement for high recognition of key features in vulnerability classification tasks. After applying max pooling to the output of each filter, the resulting feature values are concatenated in sequence, forming the final feature representation of the pooling layer. This process compresses the data and eliminates redundant features, and effectively reduces computational complexity and avoids overfitting risks. It also deeply consolidates key features of the text. This significantly enhances the discriminatory power and classification accuracy of subsequent models in detecting vulnerability features, and lays a solid foundation for constructing a precise and efficient vulnerability classification framework.

This study optimizes TextCNN by introducing multi-scale filters to better capture semantic information of varying lengths, enriching vocabulary representation with pre-trained word embeddings. Dynamic filters and residual connections effectively mitigate the vanishing gradient problem and accelerate model convergence, while the attention mechanism focuses the model on important parts of the text. Regularization and batch normalization help improve the model’s generalization ability and resistance to overfitting. These optimizations collectively make TextCNN more efficient and accurate in handling text classification tasks.

### Memristor-based neural networks

Neural networks rely on their complex structures to process information, making data-intensive problems particularly prominent in this field. The issues of the “memory wall” and “power wall” underscore the urgent need to address the high energy consumption of neural networks and improve their computational speed. This is a pressing task for the advancement of artificial intelligence algorithms. Memristors, with their unique advantages of integrated storage and computation, low power consumption, high parallelism, and high integration, align well with the needs of neural networks [24]. Researchers are focused on how to integrate memristors with neural networks to leverage their advantages and address potential problems in neural networks.

In artificial neural networks (ANNs), synapses connect neurons and are responsible for storing and transmitting information. The tunability of synaptic weights is a fundamental requirement for simulating biological synapses, and memristors are ideal for this role due to their ability to continuously change resistance under charge control and their memory function, which maintains resistance stability even after power is cut off [25]. Synaptic (weight) values need to be uniformly adjustable within a limited range. Traditional Complementary Metal-Oxide Semiconductor (CMOS) devices require dozens of components to simulate synaptic behavior, whereas memristors, with their physical properties and compact size, can achieve higher synaptic density. Common types of memristor synapses include single memristor synapses and bridge memristor synapses. Single memristor synapses use a memristor directly as a neural network synapse; the simple structure of the memristor allows it to be integrated into crossbar arrays, significantly increasing synaptic density and parallelism, enhancing computational efficiency, and reducing circuit complexity [26]. However, the use of single memristors also presents issues, such as limited resistance range, discontinuous resistance changes (with mutation points), and the inability to maintain resistance over time.

**Fig 2:**
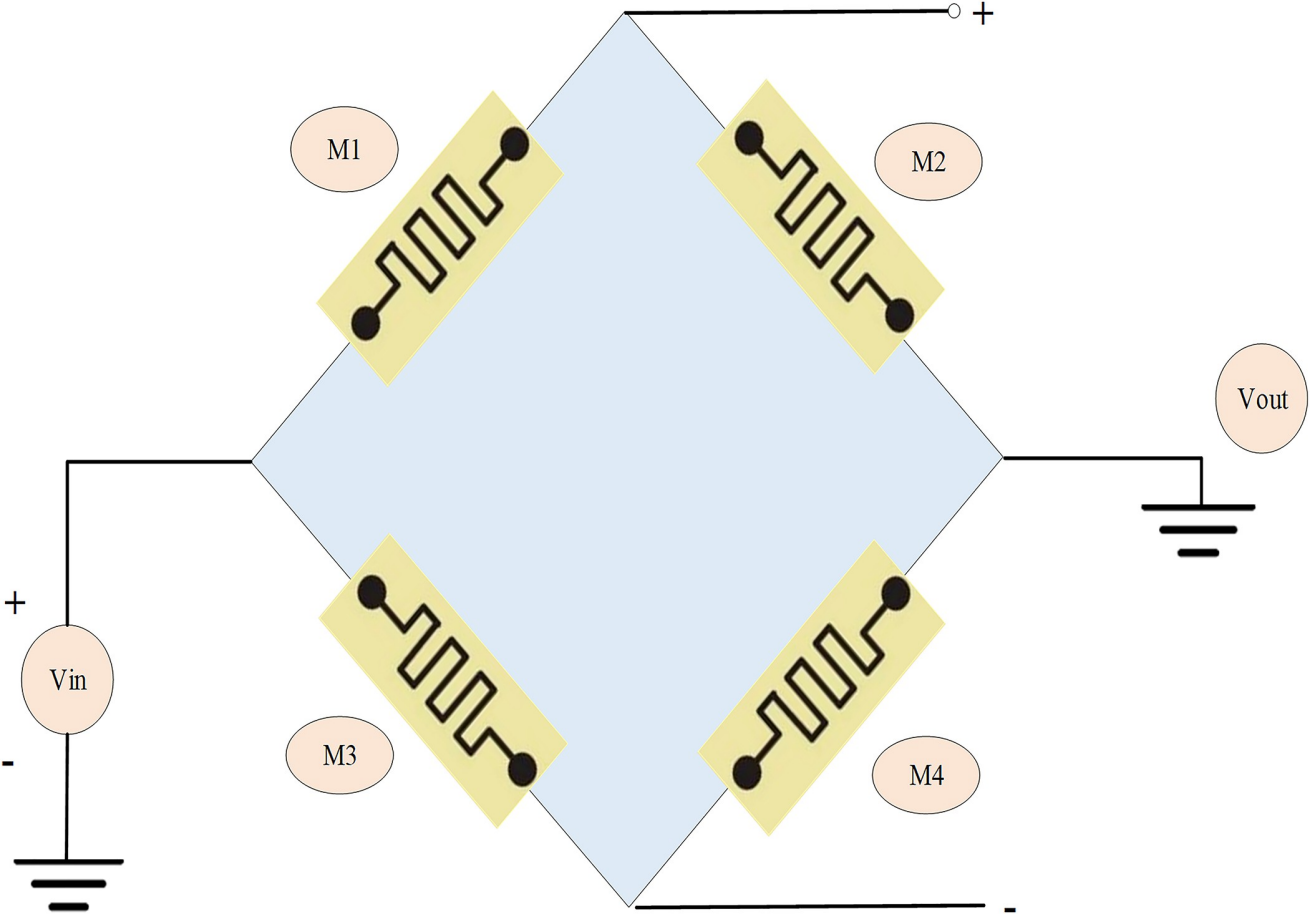
Bridge memristor synapse.

Bridge memristor synapses consist of four memristors connected in a specific polar relationship, where the output voltage depends on the voltage division among the four memristors, as shown in Fig 2.

Compared to single memristor synapses, the bridge structure can better handle external environmental impacts such as temperature drift, improving the accuracy of synaptic weight updates and reducing the Precision (P) requirements for components. However, bridge circuits require more memristors, making them less suitable for memristor arrays, thereby reducing circuit integration density. Additionally, adjusting synaptic weights requires larger pulse widths and amplitudes [27, 28]. Given the numerous and complex connections in neural networks, which demand high parallelism, single memristor synapses offer better applicability than bridge memristor synapses. Memristor Crossbar Arrays (MCA) consist of horizontal and vertical nanowires with memristors at the intersections, maximizing memristor computational parallelism and integration density. According to Ohm’s Law and Kirchhoff’s Law, the output current of an MCA is the weighted sum of column-encoded weights and input voltages, i.e., the product of the memristor conductance matrix and the input voltage vector. As an analog computation unit, MCA achieves matrix multiplication with a time complexity of O(1), which remains constant regardless of array size, enabling area and energy-efficient multiply-accumulate operations.

The primary function of neurons in the fully connected layer is to multiply input vectors by weight vectors, which aligns with the operation rules of memristor arrays; thus, it is simply a matter of mapping the neuron weight matrix onto the memristor array. Convolutional layer neurons differ from fully connected layer neurons; convolution operations involve element-wise multiplication and summation, necessitating the conversion of convolution operations into vector multiplications. By transforming the convolutional kernel into a vector with n rows and one column, and similarly transforming the corresponding submatrix in the input feature map, the convolution operation can be converted into vector multiplication. In summary, the introduction of memristors not only addresses the high energy consumption issues in neural networks but also enhances computational speed and parallel processing capabilities, driving further advancements in artificial intelligence algorithms [29].

### Model performance evaluation metrics

Model evaluation is an indispensable part of machine learning model development, aimed at validating the performance of trained models. This step is crucial for determining the best data representation and understanding the future performance of the chosen model. In machine learning, tasks are often categorized into regression, classification, and clustering, each with its unique evaluation metrics. For classification tasks, evaluation metrics may include ACC, P, R, and F1 score, which help assess the model’s performance on classification problems.

Given a test set $\left\{\left(x^{\left(1\right)},y^{\left(1\right)}),\ldots ,(x^{\left(N\right)},y^{\left(N\right)}\right)\right\}$, where the true label ***y***^(***n***)^∈{**1**,…,***C***}, the trained model ***f***(***x***; ***θ***) predicts each sample in the test set, resulting in $\left\{{\hat{y}}^{\left(1\right)},\ldots ,{\hat{y}}^{\left(N\right)}\right\}$. For class *c*, the model’s results on the test set can be categorized into True Positive (TP), False Positive (FP), True Negative (TN), and False Negative (FN) [30, 31].

TP represents the number of samples where the true class is ***c*** and the model correctly predicts the class as ***c***. This quantity is denoted by Eq (1):

$$TP_{c}=\sum_{n=1}^{N}I(y^{\left(n\right)}={\hat{y}}^{\left(n\right)}=c)$$ (1)

FP refers to the number of samples where the true class is not ***c***, but the model incorrectly predicts the sample as class ***c***. The quantity of such samples is denoted by Eq (2).


$$FP_{c}=\sum_{n=1}^{N}I(y^{\left(n\right)}\neq c\wedge {\hat{y}}^{\left(n\right)}=c)$$ (2)

TN refers to the number of samples where the true class is not ccc, and the model also predicts it as not ccc. The quantity of such samples is denoted as *TNc*. This situation generally does not require attention for class ***c***.

FN refers to the number of samples where the true class is ***c***, but the model incorrectly predicts the sample as not ***c***. The quantity of such samples is denoted by Eq (3),

$$FN_{c}=\sum_{n=1}^{N}I(y^{\left(n\right)}=c\wedge {\hat{y}}^{\left(n\right)}\neq c)$$ (3)

In Eq (3), *I* is the indicator function.

Accuracy (ACC): This is the proportion of correctly predicted samples out of the total number of samples, as shown in Eq (4).


$$ACC=\frac{1}{N}\sum_{n=1}^{N}I(y^{\left(n\right)}={\hat{y}}^{\left(n\right)})=\frac{TP+TN}{TP+TN+FP+FN}$$ (4)

Precision (P): The P for class ***c*** is the ratio of correctly predicted samples of class ***c*** to all samples predicted as class ccc. It reflects how accurate the predictions are. Samples predicted as class ***c*** can be of two types: one where class ***c*** is correctly predicted as class ***c***(***TP***_***c***_), and one where another class is incorrectly predicted as class ***c***(***FP***_***c***_).


$$P=\frac{TP_{c}}{TP_{c}+{FP}_{c}}$$ (5)

Recall (R): The R for class ***c*** is the ratio of correctly predicted samples of class ccc to all samples with the true label ***c***. It reflects how comprehensive the predictions are. For samples with the true label ***c***, the prediction can either be correct ***c***(***TP***_***c***_) or incorrect ***c***(***FN***_***c***_). The calculation of *R* is shown in Eq (6).


$$R=\frac{TP_{c}}{TP_{c}+FN_{c}}$$ (6)

F1 Score (F1) is the harmonic mean of *P* and *R*, used for a comprehensive evaluation of the classification model’s performance, especially in imbalanced datasets.


$$F1=\frac{2∙P∙R}{P+R}$$ (7)

## Experimental data design

This study uses a vulnerability dataset from the National Vulnerability Database (NVD) as the basis for experimental data. The dataset is presented in JSON format, with a time span from 2002 to May 2024. It contains rich vulnerability information, with each record consisting of a unique CVE-ID, a detailed description text, and the corresponding CWE-ID. During the data preprocessing phase, the vulnerabilities obtained from the NVD are numbered based on the CVE-ID (e.g., "CVE-2008-0988"). This numbering system indicates that the vulnerability is the 988th one discovered in 2008 and belongs to CWE-89, the SQL injection vulnerability type. All vulnerability descriptions are provided in English. To effectively mitigate the potential adverse impact of sample imbalance on model construction and analysis, this study conducts the following in-depth preprocessing operations on the dataset: First, a comprehensive and detailed statistical analysis is performed on all vulnerability categories in the dataset to accurately determine the distribution of each category’s occurrence. Based on these statistical results, eight vulnerability categories that are both sufficiently represented and frequently appear in practical network security scenarios are carefully selected. Then, for each record in the dataset, the corresponding vulnerability category based on its CWE-ID is used to precisely extract all records belonging to the eight selected categories. In this way, a targeted dataset specifically for subsequent model training and analysis is constructed. This approach ensures the validity and representativeness of the data, providing a solid foundation for accurate vulnerability analysis and robust model construction. It also ensures that the model is trained in a relatively balanced and representative data environment, effectively improving the model’s accuracy and generalization ability to better address practical vulnerability research and analysis tasks. The categories and distribution for each CWE-ID are shown in Table 1.

**Table 1:** Vulnerability categories corresponding to each CWE-ID.

CWE-ID	Vulnerability categories	distribution
CWE-79	Cross-site scripting	13749
CWE-119	Buffer error	12780
CWE-200	Information leakage	7552
CWE-89	SQL injection	6012
CWE-22	Path traversal	3302
CWE-352	Cross-site request forgery	2699
CWE-125	Out-of-bounds read	2630
CWE-310	Encryption issues	2537

In addition, public datasets play a crucial role in vulnerability analysis and model construction within the field of cybersecurity research. Several publicly available datasets related to this study each have their unique features and value. The Devign dataset collects functions from specific large-scale open-source projects, such as LinuxKernel and Qemu, and annotates them manually. Its function-level annotations and relatively balanced distribution of vulnerable and non-vulnerable functions provide researchers with valuable material for vulnerability feature extraction and model training at the function level. The Reveal dataset focuses on real-world vulnerabilities in the Chromium and Debian projects. This dataset, based on vulnerabilities reported by actual developers and users, helps make the study more aligned with real-world vulnerability occurrence and discovery scenarios, aiding the construction of more practical vulnerability detection models. The BigVul dataset is large in scale and covers numerous open-source GitHub projects, containing rich information about functions and code lines. Its detailed CWE classifications and line-number-level annotations facilitate in-depth exploration of the distribution patterns and feature models of vulnerabilities at the code line level. Built on BigVul, the Real-Vul dataset selects key projects and applies data augmentation techniques to further enhance the usability and representativeness of the data, making the dataset more adaptable to the complex and dynamic nature of real-world vulnerabilities. The SARD+NVD dataset integrates resources from the National Vulnerability Database and the Software Assurance Reference Dataset, covering both real-world programs and synthetically created ones. Its multi-level annotations and multiple data formats offer flexible options for research needs, whether at the function level or line-number level, providing valuable data support for vulnerability studies. JulietC/C++1.3.1, created by the authoritative NIST, covers a wide range of CWE vulnerability types through extensive test cases, offering a comprehensive and standardized reference for vulnerability research in C/C++ languages. The PRIMEVUL dataset excels not only in the number of functions and CWE coverage but also in its innovative data annotation techniques and strict data processing strategies. These aspects are beneficial for improving data quality and advancing vulnerability research in a more precise and efficient direction. Overall, these public datasets complement each other, offering rich data resources and research foundations for this study and the broader field of cybersecurity vulnerability research. They contribute to the continuous development and refinement of vulnerability analysis techniques and models.

## Analysis of computer vulnerability intelligent classification results

### Evaluation of MNN model classification performance

**Fig 3:**
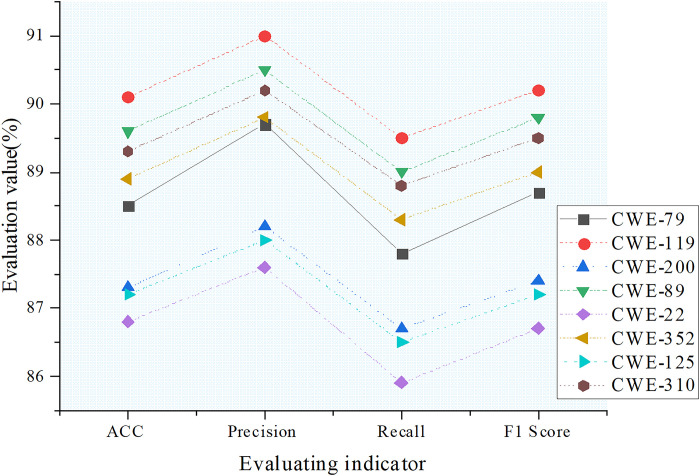
Evaluation of MNN model classification performance across different vulnerability categories.

The classification performance of the MNN model across different vulnerability categories is shown in Fig 3.

In Fig 3, the MNN model demonstrates relatively stable and efficient classification performance across various vulnerability categories. For example, in the CWE-119 category, the model achieves an accuracy of 90.1%, precision of 91.0%, recall of 89.5%, and an F1 score of 90.2%. This indicates that the MNN model exhibits high accuracy and comprehensiveness in detecting and classifying buffer overflow errors. The model performs similarly well in other categories, such as CWE-79 and CWE-89, with accuracies of 88.5% and 89.6%, while maintaining high precision and recall values. Overall, the MNN model excels in accuracy (ACC), precision (P), recall (R), and F1 score assessments, with an average accuracy of 89.5%, average precision of 90.2%, average recall of 88.7%, and average F1 score of 89.4%. These results highlight its potential and effectiveness in network security management. These findings not only confirm the model’s ability to accurately identify various vulnerabilities but also provide solid data support for its practical application. However, a deeper analysis of the model’s performance reveals certain limitations. Although the model performs well in most categories, there is still room for improvement in some specific vulnerability categories. For instance, when handling vulnerability types with complex boundary conditions, the model may occasionally misclassify them. For vulnerabilities with atypical feature representations, the MNN model might fail to accurately classify them due to insufficient understanding of the features. Furthermore, when faced with vulnerability categories that have subtle feature differences between classes, the model’s discriminative ability may be challenged, leading to a slight decrease in classification accuracy. To address these potential issues, future work could consider further optimizing the model structure and enhancing its ability to handle complex boundary conditions and subtle feature differences. By introducing more targeted feature engineering techniques, the model’s ability to express features of various vulnerabilities could be improved, increasing its classification accuracy in the complex and dynamic real-world network environment. This would ensure that the model maintains stable and reliable performance across different scenarios, better meeting the practical needs of network security management.

**Fig 4:**
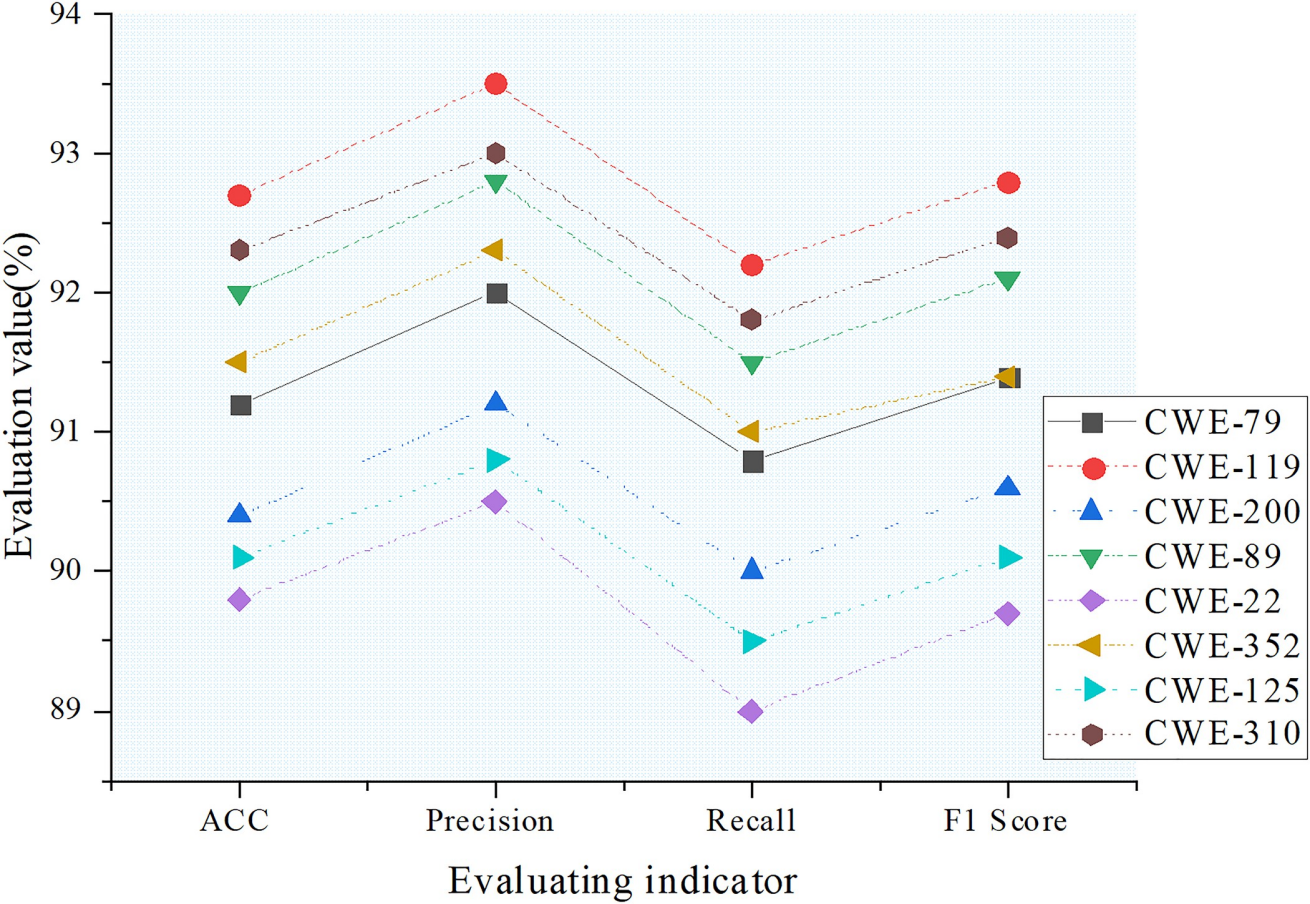
Evaluation of the improved TCNN model’s classification performance across different vulnerability categories.

The performance evaluation of the Temporal Convolutional Neural Network (TCNN) model across different vulnerability categories is shown in Fig 4.

In Fig 4, the improved TCNN model demonstrates significant enhancement and excellent performance in classification tasks across various vulnerability categories. For example, in the CWE-119 category, the model achieved an ACC of 92.7%, P of 93.5%, R of 92.2%, and an F1 score of 92.8%. These metrics indicate that the model has a high level of accuracy and comprehensiveness in detecting and identifying buffer overflow errors. In other categories, such as CWE-79 and CWE-89, the improved TCNN model also reached high performance levels, showing accuracies of 91.2% and 92.0%, and F1 scores of 91.4% and 92.1%, respectively, demonstrating its consistency and stability across various vulnerability classification tasks. Overall, the improved TCNN model, through structural optimization and parameter adjustments, has effectively enhanced its application performance in network security management, providing robust technical support and solutions for vulnerability classification and security defense.

### Comparison analysis of different models

**Fig 5:**
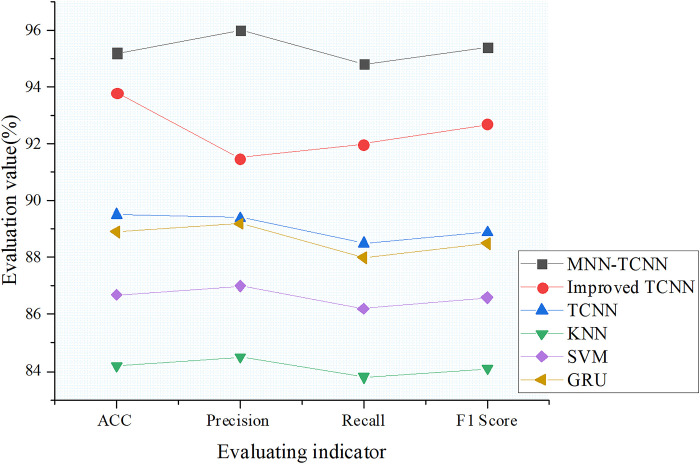
Model comparison results.

This section compares the performance of the MNN-TextCNN, improved TCNN, TCNN, K-Nearest Neighbors (KNN), Support Vector Machine (SVM), and Gated Recurrent Unit (GRU) models in vulnerability classification tasks. The comparison results are shown in Fig 5.

In the model performance comparison shown in Fig 5, the standalone TCNN model achieves an accuracy of 89.5%, precision of 89.4%, recall of 88.5%, and an F1 score of 88.9%. This model adopts the conventional CNNs architecture, where the convolutional layers use kernels to extract text features. The kernel size and other parameter settings determine the scale at which vulnerability features are extracted. In the vulnerability classification task, it can handle the diversity of vulnerability data to some extent. However, due to the model structure not being deeply optimized for vulnerability classification, such as not fully considering the complex relationships between features of different vulnerability types, the efficiency of feature extraction and utilization is limited, ultimately affecting overall performance. The improved TCNN model shows significant performance improvement, with an accuracy of 93.8%, precision of 91.5%, recall of 92.0%, and an F1 score of 92.7%. It is optimized in structure, introducing multi-scale filters that better capture semantic information of different lengths, thereby strengthening the model’s ability to express vulnerability features. The use of dynamic filters and residual connections effectively mitigates the vanishing gradient problem and accelerates model convergence, leading to noticeable improvements across all metrics. The MNN-TCNN model performs the best, with an accuracy of 95.2%, precision of 96.0%, recall of 94.8%, and an F1 score of 95.4%. This model integrates the advantages of MNN and the improved TCNN model. MNN simulates the memory effect of biological neurons and can capture complex nonlinear relationships in vulnerability data, providing richer feature information for TCNN. The improved TCNN further optimizes feature extraction and classification capabilities. The collaboration between the two models enables more precise identification of various vulnerability features, enhancing classification accuracy and comprehensiveness when dealing with complex vulnerability data. In comparison, the KNN and SVM models perform slightly worse. The KNN model classifies based on sample distance, but its distance measurement method struggles to accurately reflect sample similarity in high-dimensional, complex vulnerability data, and it has high computational costs. The SVM model encounters challenges when processing large-scale, non-linearly separable vulnerability data, as selecting kernel functions and adjusting parameters is complex, and the model is prone to getting stuck in local optima, which affects classification performance. The KNN model achieves an accuracy of 84.2%, precision of 84.5%, recall of 83.8%, and an F1 score of 84.1%. The SVM model has an accuracy of 86.7%, precision of 87.0%, recall of 86.2%, and an F1 score of 86.6%. The GRU model achieves an accuracy of 88.9%, precision of 89.2%, recall of 88.0%, and an F1 score of 88.5%, placing it between the TCNN and improved TCNN models. As a recurrent neural network, GRU has certain advantages in processing sequential data, but in the vulnerability classification task, its efficiency in extracting and utilizing text features is lower than that of CNNs. It is unable to fully explore both local and global features in vulnerability descriptions, limiting its performance improvement. In summary, the MNN-TCNN model performs exceptionally well in the vulnerability classification task, followed by the improved TCNN model. These results provide key references for selecting vulnerability classification models and can help researchers and practitioners choose the appropriate model for vulnerability classification based on specific needs and data characteristics, ultimately enhancing network security management effectiveness.

This study plays a key role in multiple aspects of practical applications. In the field of network security management, there are challenges arising from the increasing number and complexity of vulnerabilities and the limitations of traditional methods. The intelligent classification system, combining MNN and the improved TCNN, has profound significance. The system can accurately classify vulnerability data, with MNN simulating the memory effect of biological neurons to capture complex nonlinear relationships, and the optimized TCNN exhibiting excellent performance in classifying different vulnerability categories. The integrated system achieves an accuracy of 95.2%, significantly enhancing detection and classification capabilities while effectively reducing false positive rates, such as accurately identifying common vulnerabilities like CWE-119. In practical network operations and maintenance, the system helps administrators detect and address vulnerabilities promptly, reduce attack risks, and ensure the stable operation of systems, safeguarding data and service continuity and integrity. For security companies, it provides core technical support for developing efficient vulnerability management tools and enhancing product competitiveness. It also strengthens network security defenses in industries such as finance, healthcare, and industrial control, while protecting critical information assets and business processes. This approach comprehensively improves the level of network security defense and management efficiency.

## Conclusion

In the study of vulnerability classification tasks, this study demonstrates the superiority of the MNN-TCNN model across multiple evaluation metrics, including ACC, P, R, and F1 score. Specifically, the MNN-TCNN model achieves an ACC of 95.2%, P of 96.0%, R of 94.8%, and an F1 score of 95.4%, clearly outperforming other comparison models. Although the improved TCNN model is slightly lower than the MNN-TCNN model, it still performs excellently across all metrics, showing its stability and efficiency in vulnerability classification tasks. This study not only validates the synergistic effects of MNN and TCNN models in complex network environments but also explores effective strategies for enhancing TCNN model performance through structural optimization and parameter adjustments. These research findings are significant for advancing the technology of computer vulnerability classification and provide substantial technical support for optimizing future network security management systems. Overall, the study highlights the superiority of deep learning models such as MNN-TCNN in vulnerability classification tasks and underscores the importance of integrating intelligent classification systems into network security management. Future research directions could further explore the application and optimization of these models in more complex and dynamic network security environments and how to implement these technologies in practical network security systems to enhance their capability and efficiency in countering vulnerability attacks.

## Supporting information

S1 Data (XLSX)

## References

[1] Aslan Ö, Aktuğ S S, Ozkan-Okay M, Yilmaz A A, Akin E. A comprehensive review of cyber security vulnerabilities, threats, attacks, and solutions. Electronics, 2023; 12(6): 1333.

[2] Alanazi M, Mahmood A, Chowdhury M J M. SCADA vulnerabilities and attacks: A review of the state-of-the-art and open issues. Computers & Security, 2023; 125(8): 103028.

[3] Hulayyil S B, Li S, Xu L. Machine-learning-based vulnerability detection and classification in internet of things device security. Electronics, 2023; 12(18): 3927.

[4] Muhammad S, Lawan A M, Muhammad A. Management of vulnerabilities in cyber security. Journal homepage: https://gjrpublication.com/gjrecs, 2023; 3(02): 43.

[5] Filus K, Domańska J. Software vulnerabilities in TensorFlow-based deep learning applications. Computers & Security, 2023; 124: 102948.

[6] Liu X, Tang T, Ding N. Social network sentiment classification method combined Chinese text syntax with graph convolutional neural network. Egyptian Informatics Journal, 2022; 23(1): 1–12.

[7] Wang Q, Gao Y, Ren J, Zhang B. An automatic classification algorithm for software vulnerability based on weighted word vector and fusion neural network. Computers & Security, 2023; 126(7): 103070.

[8] Wan B, Xu C, Koo J. Exploring the effectiveness of web crawlers in detecting security vulnerabilities in computer software applications. International Journal of Informatics and Information Systems, 2023; 6(2): 56–65.

[9] Do Xuan C, Mai D H, Thanh M C, Van Cong B. A novel approach for software vulnerability detection based on intelligent cognitive computing. The Journal of Supercomputing, 2023; 79(15): 17042–17078.

[10] Napier K, Bhowmik T, Wang S. An empirical study of text-based machine learning models for vulnerability detection. Empirical Software Engineering, 2023; 28(2): 38.

[11] Kuehn P, Relke D N, Reuter C. Common vulnerability scoring system prediction based on open source intelligence information sources. Computers & Security, 2023; 131(20): 103286.

[12] Silvestri S, Islam S, Papastergiou S, Tzagkarakis C, Ciampi M. A machine learning approach for the NLP-based analysis of cyber threats and vulnerabilities of the healthcare ecosystem. Sensors, 2023; 23(2): 651. doi: 10.3390/s23020651 36679446 PMC9866080

[13] Kekül H, Ergen B, Arslan H. Estimating vulnerability metrics with word embedding and multiclass classification methods. International Journal of Information Security, 2024; 23(1): 247–270.

[14] Fu M, Tantithamthavorn C, Le T, Kume Y, Nguyen V, Phung D, et al. Aibughunter: A practical tool for predicting, classifying and repairing software vulnerabilities. Empirical Software Engineering, 2024; 29(1): 4.

[15] Chen H, Zhang Z, Huang S, Hu J, Ni W, Liu J. TextCNN-based ensemble learning model for Japanese text multi-classification. Computers and Electrical Engineering, 2023; 109(8): 108751.

[16] Huo Z, Fan Y, Huang Y. A communication-efficient federated text classification method based on parameter pruning. Mathematics, 2023; 11(13): 2804.

[17] Pan M, Wu P, Zou Y, Ruan C, Zhang T. An automatic vulnerability classification framework based on BiGRU-TextCNN. Procedia Computer Science, 2023; 222(3): 377–386.

[18] Kong Y, Huang N, Deng H, Feng J, Liang X, Lv W, et al. Text classification in fair competition law violations using deep learning. Frontiers in Applied Mathematics and Statistics, 2023; 9(3): 1177081.

[19] Qin Y, Irshad A. Research on the evaluation method of English textbook readability based on the TextCNN model and its application in teaching design. PeerJ Computer Science, 2024; 10(2): e1895. 10.7717/peerj-cs.1895 PMC10909215 38435600

[20] Wang Z. Deep learning based text classification methods. Highlights in Science, Engineering and Technology, 2023; 34(5): 238–243.

[21] Wang Z, Wang L, Huang C, Sun S, Luo X. BERT-based Chinese text classification for emergency management with a novel loss function. Applied Intelligence, 2023; 53(9): 10417–10428.

[22] Malhotra R, Vidushi. Text mining based an automatic model for software vulnerability severity prediction. International Journal of System Assurance Engineering and Management, 2024; 1(3): 1–19.

[23] Aguirre F, Sebastian A, Le Gallo M, Song W, Wang T, Yang J J, et al. Hardware implementation of memristor-based artificial neural networks. Nature Communications, 2024; 15(1): 1974. doi: 10.1038/s41467-024-45670-9 38438350 PMC10912231

[24] Cao Z, Sun B, Zhou G, Mao S, Zhu S, Zhang J, et al. Memristor-based neural networks: a bridge from device to artificial intelligence. Nanoscale Horizons, 2023; 8(6): 716–745. doi: 10.1039/d2nh00536k 36946082

[25] Yi S I, Kendall J D, Williams R S, Kumar S. Activity-difference training of deep neural networks using memristor crossbars. Nature Electronics, 2023; 6(1): 45–51.

[26] Lin H, Wang C, Yu F, Hong Q, Xu C, Sun Y. A triple-memristor Hopfield neural network with space multistructure attractors and space initial-offset behaviors. IEEE Transactions on Computer-Aided Design of Integrated Circuits and Systems, 2023; 42(12): 4948–4958.

[27] Ye L, Gao Z, Fu J, Ren W, Yang C, Wen J, et al. Overview of memristor-based neural network design and applications. Frontiers in Physics, 2022; 10(1): 839243.

[28] Wang C, Liang J, Deng Q. Dynamics of heterogeneous Hopfield neural network with adaptive activation function based on memristor. Neural Networks, 2024; 178(12): 106408. doi: 10.1016/j.neunet.2024.106408 38833751

[29] Jeon K, Ryu J J, Im S, Seo H K, Eom T, Ju H, et al. Purely self-rectifying memristor-based passive crossbar array for artificial neural network accelerators. Nature Communications, 2024; 15(1): 129. doi: 10.1038/s41467-023-44620-1 38167379 PMC10761713

[30] Bonnet D, Hirtzlin T, Majumdar A, Dalgaty T, Esmanhotto E, Meli V, et al. Bringing uncertainty quantification to the extreme-edge with memristor-based Bayesian neural networks. Nature Communications, 2023; 14(1): 7530. doi: 10.1038/s41467-023-43317-9 37985669 PMC10661910

[31] Ma M L, Xie X H, Yang Y, Li Z J, Sun Y C. Synchronization coexistence in a Rulkov neural network based on locally active discrete memristor. Chinese Physics B, 2023; 32(5): 058701.

